# A Multi-Faceted Digital Health Solution for Monitoring and Managing Diabetic Foot Ulcer Risk: A Case Series

**DOI:** 10.3390/s24092675

**Published:** 2024-04-23

**Authors:** Emily Matijevich, Evan Minty, Emily Bray, Courtney Bachus, Maryam Hajizadeh, Brock Liden

**Affiliations:** 1 Orpyx Medical Technologies, Inc., Calgary, AB T2G 1M8, Canada; 2 Cumming School of Medicine, University of Calgary, Calgary, AB T2N 4N1, Canada; 3 Cutting Edge Research LLC, Circleville, OH 43113, USA

**Keywords:** diabetic foot ulcers (DFU), remote patient monitoring (RPM), sensor-based monitoring, diabetes complications, neuropathy, plantar pressure, wearables, health technology, temperature monitoring

## Abstract

Introduction: Diabetic foot ulcers (DFU) are a devastating complication of diabetes. There are numerous challenges with preventing diabetic foot complications and barriers to achieving the care processes suggested in established foot care guidelines. Multi-faceted digital health solutions, which combine multimodal sensing, patient-facing biofeedback, and remote patient monitoring (RPM), show promise in improving our ability to understand, prevent, and manage DFUs. Methods: Patients with a history of diabetic plantar foot ulcers were enrolled in a prospective cohort study and equipped with custom sensory insoles to track plantar pressure, plantar temperature, step count, and adherence data. Sensory insole data enabled patient-facing biofeedback to cue active plantar offloading in response to sustained high plantar pressures, and RPM assessments in response to data trends of concern in plantar pressure, plantar temperature, or sensory insole adherence. Three non-consecutive case participants that ultimately presented with pre-ulcerative lesions (a callus and/or erythematous area on the plantar surface of the foot) during the study were selected for this case series. Results: Across three illustrative patients, continuous plantar pressure monitoring demonstrated promise for empowering both the patient and provider with information for data-driven management of pressure offloading treatments. Conclusion: Multi-faceted digital health solutions can naturally enable and reinforce the integrative foot care guidelines. Multi-modal sensing across multiple physiologic domains supports the monitoring of foot health at various stages along the DFU pathogenesis pathway. Furthermore, digital health solutions equipped with remote patient monitoring unlock new opportunities for personalizing treatments, providing periodic self-care reinforcement, and encouraging patient engagement—key tools for improving patient adherence to their diabetic foot care plan.

## 1. Introduction

Diabetic foot ulcers (DFU), or wounds on the foot, are a devastating and complex complication of diabetes [1]. DFU development can stem from mechanical or ischemic factors [2]. With the mechanical pathway of DFU development, peripheral neuropathy and loss of protective sensation (LOPS) interfere with an individual’s ability to sense and offload harmful sustained plantar pressures [2,3]. Additional risk factors that may contribute to abnormal plantar pressures include loss of intrinsic foot muscles, changes in foot shape, foot deformities, and altered gait and posture biomechanics [2,3,4]. Abnormal plantar pressures can result in callus formation, inflammation, and tissue damage or ulcers extending to the subcutaneous tissue or deeper [2].

Of people living with diabetes, 34% are likely to develop a DFU during their lifetime [5]. DFU recurrence rates are high, with an estimated 40% of ulcers recurring within the first year of healing [5]. Over one-third of DFUs result in lower extremity amputation (LEA) of the toes, the entire foot, or the lower leg [6]. The consequences of DFUs extend beyond amputation and place patients at risk for numerous other adverse events such as falls, fractures, reduced mobility, frailty, and mortality [7].

Fortunately, it is estimated that 75% of DFUs are preventable using established foot care methods and are treatable when detected early [6,8]. The International Working Group on the Diabetic Foot (IWDGF) offers evidence-based guidelines on the prevention and management of DFUs as part of integrative foot care best practices [9]. However, there are numerous challenges with achieving these recommendations and adhering to the guidelines (Table 1).

Several strategies have been proposed to address the challenges of established foot care guidelines (Table 1). First, real-time plantar pressure offloading through biofeedback (e.g., via an intelligent insole system [17]) has been suggested as a strategy to compensate for the loss of plantar sensation due to diabetic peripheral neuropathy. Active plantar pressure offloading is believed to support DFU prevention by reducing the periods of elevated, repetitive, and undetected plantar pressures that can cause cumulative tissue mechanical stress [4,18] or exceed capillary perfusion pressure across a time window capable of causing tissue injury [19,20].

Second, temperature monitoring has been proposed as a strategy to identify an inflammatory response as a preliminary sign of tissue damage [21]. Reduced ambulatory activity in response to “hotspot” detection (e.g., contralateral temperature asymmetries > 2.2 °C) is believed to provide the offloading necessary to reduce inflammation and DFU risk. Protocols typically involve contacting a care provider when the hotspots are detected. While skin temperature monitoring was initially enabled by handheld daily temperature measurements with infrared dermal thermometers [21], sensor-based digital health technologies (e.g., sensory socks [22], smart mats [23], etc.) have been developed to facilitate improved consistency and ease of measurement.

Third, strategies have been suggested to encourage adherence to the diabetic foot health management care plan. Several technologies have been proposed to monitor prescribed footwear use or adherence to other aspects of the care plan [15]. Integrating these data into remote patient monitoring (RPM) systems offers objective insights to encourage patient engagement, including personalized structured education and reinforcement of self-care practices [24].

While these individual strategies have shown the potential for reducing DFU risk, a multi-faceted digital health solution (i.e., fusion of multimodal sensing, direct patient biofeedback, and RPM) may better align with the multifactorial causal pathway of DFU formation. However, such holistic strategies for reducing DFU risk are underexplored, and the compounding benefits are unknown [24,25].

In this case series, we present patient narratives, physiologic data, and RPM engagement from a multi-faceted digital health solution that highlights both a multimodal approach to diabetic foot monitoring (plantar pressure, plantar temperature, activity, and device adherence monitoring via a sensory insole), as well as multiple data-driven action pathways (direct patient biofeedback and remote patient monitoring). The purpose of this case series is to explore how a multi-faceted digital health solution may enable and reinforce established diabetic foot care guidelines and evaluate how such holistic solutions can improve our ability to understand, prevent, and manage patients at risk for DFUs.

## 2. Materials and Methods

A prospective cohort study was conducted at a single office-based podiatry clinic in Ohio, USA. Three non-consecutive case participants who presented with pre-ulcerative lesions (a callus and/or erythematous area on the plantar surface of the foot) during the study were selected for presentation in this case series. Presented cases were selected as they were illustrative of situations that may arise in the clinical management of the diabetic foot. The study received Institutional Review Board (IRB) approval through WCG IRB (20220828). Informed consent was obtained from all patients in the study. Patients who had type 1 or 2 diabetes, peripheral neuropathy, and a history of a previous plantar foot ulcer were candidates for recruitment. Patients with active ulcer(s) or other open chronic wounds, presence of severe vascular disease, history of a non-neuropathic foot ulcer, or a serious underlying balance issue were excluded.

**Figure 1:**
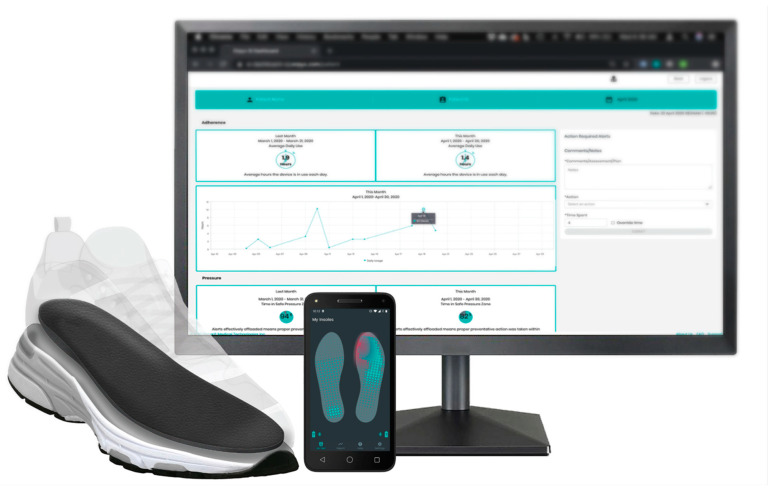
The Sensory Insole System (Orpyx^®^ Sensory Insole System, Orpyx Medical Technologies Inc., Calgary, AB, Canada) includes custom-milled insoles that are placed into the patient’s shoes; a patient-facing app that provides real-time pressure feedback, step count, and wear time information; and a web-based dashboard accessed by a remote patient monitoring nurse and provider to review the data collected from the sensory insoles.

All three case participants were provided with custom sensory insoles (Orpyx^®^ Sensory Insoles, Orpyx Medical Technologies Inc., Figure 1) to track, analyze, and trend plantar pressure, plantar temperature, step count, and usage data as they went about their daily activities. Participants wore the sensory insole system for at least 8 months (chosen arbitrarily based on the amount of sensory insole usage at the time of writing) and were instructed to wear the insoles in standardized diabetic footwear for a minimum of 4.5 h per day [17]. The digital health solution included adjunct RPM, provided through the in-house RPM service at Orpyx Medical Technologies. While the Orpyx Sensory Insoles were used for patient monitoring in this study, there is great flexibility in selecting a sensor-based technology for integration with RPM, with the goal of balancing effectiveness and practicality of the digital health solution for a specific use case [25].

### 2.1. Plantar Pressure

**Figure 2:**
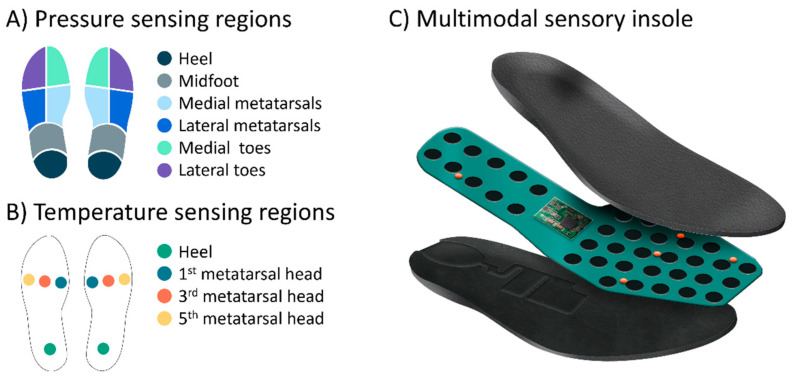
Insole sensory regions. (**A**) Pressure-sensing regions. (**B**) Temperature-sensing regions. (**C**) Multimodal sensory insole system containing pressure, temperature, and motion sensors embedded in a custom orthotic. Large black circles illustrate the array of discrete force sensitive resistors (FSR) to record plantar pressure. Small orange circles illustrate the five temperature sensors located beneath the high-risk bony prominences in the foot (metatarsal heads 1, 3, and 5, the heel, and the big toe). At the time of the study, temperature asymmetry monitoring was inactive at the big toe. The inertial measurement unit (IMU) is embedded in the electronics chip in the center of the insole.

Each insole (depending on the insole size) comprises an array of 22–37 discrete force sensitive resistors (FSR) to record plantar pressure. Each FSR element operates as a switch at pressures greater than 35–50 mmHg, a threshold chosen based on estimates of capillary perfusion pressure at the foot [17]. When 95% or greater of the insole pressure measurements exceed the calibrated pressure threshold over a 15 min sliding time window, the sensor would be marked as being in a “high-pressure state” and the app-based display would provide real-time patient-facing biofeedback for pressure offloading [17]. The sensory insole technology has been shown to reliably detect pressures above a calibrated pressure threshold for most sensor locations [26]. For RPM review, high-pressure states were distilled to six anatomical foot regions per insole (Figure 2A). When any combination of foot regions was in a high-pressure state for greater than 40% of usage time for a day, a warning indicator was generated for the RPM review.

### 2.2. Temperature

Each insole consisted of 5 temperature sensors located beneath high-risk bony prominences in the foot (metatarsal heads 1, 3, and 5, the heel, and the big toe, Figure 2B). At the time of the study, temperature asymmetry monitoring was inactive at the big toe. The temperature measured by the sensors are accurate within 0.6 °C of a reference standard (unpublished data), similar accuracy to other wearable plantar temperature monitoring solutions [22]. Temperature was summarized as the daily contralateral temperature difference between left and right corresponding foot locations (temperature asymmetry). Temperature was also summarized as the daily ipsilateral temperature difference between a foot location and the average of all foot locations on the same foot. At the time of this study, for consistency with escalation processes in previous randomized clinical trials that examined temperature asymmetries [21], measurements were evaluated at a single time point. When two consecutive daily temperature difference measurements exceeded a 2.2 °C threshold, a warning indicator was generated for RPM review. The product did not include any real-time patient-facing biofeedback triggered by temperature asymmetries.

### 2.3. Step-Count, Daily Insole Usage, and Adherence Monitoring

An inertial measurement unit (IMU) was embedded in the sensory insole to record foot motion. Daily insole usage was estimated as the duration of daily data collection triggered by foot motion. A custom step-count algorithm was used to report daily step count. Daily usage and step count were used to contextualize patient behavior and monitor adherence. Given that the sensory insole was placed in the patient’s diabetic footwear, sensory insole usage also served as a surrogate measure of adherence to wearing the diabetic footwear. If no usage was detected for a period of three consecutive days, an adherence warning indicator was generated for RPM review.

### 2.4. Remote Monitoring and Case Escalation

Participants were remotely monitored by a U.S.-based qualified healthcare professional who routinely reviewed the data collected by the sensory insoles published to a dashboard (Figure 1). Data trends of concern generated warning indicators for the RPM nurse to review. Participants were contacted by the RPM nurse based on a mutually agreed upon escalation protocol or in accordance with their clinical judgement. Contact with patients typically entailed a discussion of the data trend of concern, remote assessment of the patient’s feet, if possible, and coaching and education on reducing risk factors through foot care best practices. When a significant or persistent data trend of concern emerged, or when RPM engagement with the patient revealed a potential concern, the patient was escalated to the referring clinician and an in-person clinic visit was scheduled at their discretion. The type of RPM engagement (successful phone call, as defined by having a patient interaction, vs. data review only) and duration was automatically tracked in the dashboard.

## 3. Results

### 3.1. Case 1

**Figure 3:**
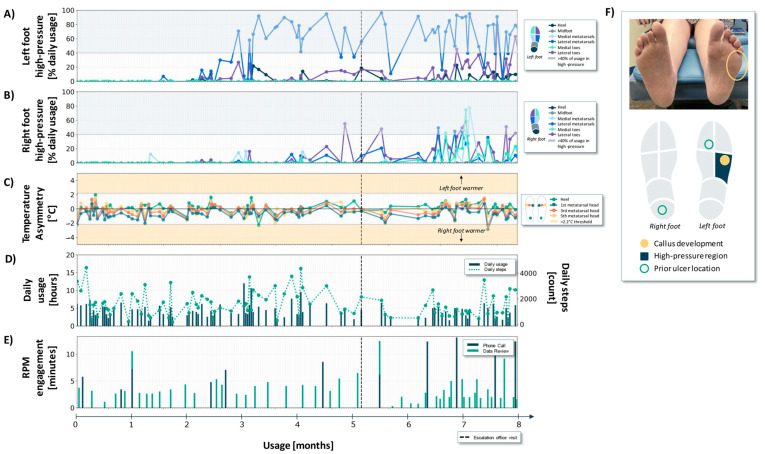
Pressure, temperature, daily usage, and RPM engagement metrics over an 8-month period for Case 1. (**A**,**B**) High-pressure states, expressed as a percentage of daily usage, for the different regions of the left and right foot, respectively. Any regions falling in blue-shaded area were in a high-pressure state for more than 40% of the usage time. (**C**) Temperature asymmetries. Data points in the upper and lower yellow regions indicate one foot is at least 2.2 °C warmer than the other. (**D**) Daily usage and step count. (**E**) RPM engagement phone calls and data review. It is possible that more than one RPM engagement occurred in a single day. (**F**) Photographs of the plantar surface of the patient’s feet highlighting areas of callus development seen in-clinic, alongside a foot map summarizing high-pressure regions and prior ulcer locations.

Case 1 is a 49-year-old female with a 20-year history of poorly controlled type 2 diabetes, and a history inclusive of psoriatic arthritis. They demonstrated complete loss of protective sensation bilaterally on the basis of 5.07 monofilament testing and had a history of recurrent DFUs on the left second toe and right heel (Figure 3F). The patient had forefoot varus in their right foot as well as a triple arthrodesis, which resulted in a fused and locked subtalar joint, and angular alignment of the right heel.

During the 8-month usage period, the patient wore the sensory insoles for an average of 4.3 (±1.9) h per day with an average step count of 1583 (±917) steps (Figure 3D).

Between months 3 and 5, the patient experienced consistent high-pressures in the left foot lateral metatarsal region (Figure 3A). The lateral left foot high-pressures in this time range are consistent with a compensatory loading strategy due to the right foot deformity [27]. In view of these sustained high-pressure patterns, the RPM nurse maintained frequent engagement with the patient and periodically monitored the sensory insole data to ensure that there were no other data trends of concern (Figure 3E). Prior to the patient’s scheduled clinic visit, the RPM nurse engaged with the patient and their data 32 times (7 unique phone calls and 25 unique data review sessions) (Figure 3E, months ~0–5). During the phone calls, the patient did not report any visible abnormalities or concerns on self-exam despite the persistent high-pressures measured by the sensory insoles.

In communication with the provider about the pressure data trend of concern, in-person patient assessment was deferred to their scheduled follow-up. During that in-clinic visit (vertical black dashed line in Figure 3A–E), the patient presented with a callus and cracking on the left lateral foot underneath the fifth metatarsal head. The clinician addressed the callus by adding a lateral post to the left insole (a strip of tapered material on the lateral side of the insole running from the heel past the fifth metatarsal head).

After the clinic visit and insole modification (Figure 3, months ~5–6), the observed plantar pressures on the left and right feet did not change significantly. Subsequently, sensory insole data revealed continued high-pressure on the left lateral foot, and a trending increase in high-pressure on the right foot (Figure 3A,B, months ~6–8).

This patient did not generate many temperature asymmetries in the 8-month usage window. Only two non-consecutive data days with a contralateral temperature difference exceeding a 2.2 °C threshold were detected (Figure 3C).

### 3.2. Case 2

Case 2 is a 60-year-old male with a history of type 2 diabetes, severe peripheral neuropathy, and renal transplant for end stage renal disease (ESRD) from diabetic nephropathy. The patient also had a history of a cerebrovascular accident (CVA) with a resulting left lower extremity motor deficit, but they were ambulating independently at enrollment. They had a history of previous DFUs on the right fifth metatarsal head, right lateral foot, and left first toe. They also had a history of a DFU to the right first toe, and subsequent right first toe amputation (Figure 3F).

**Figure 4:**
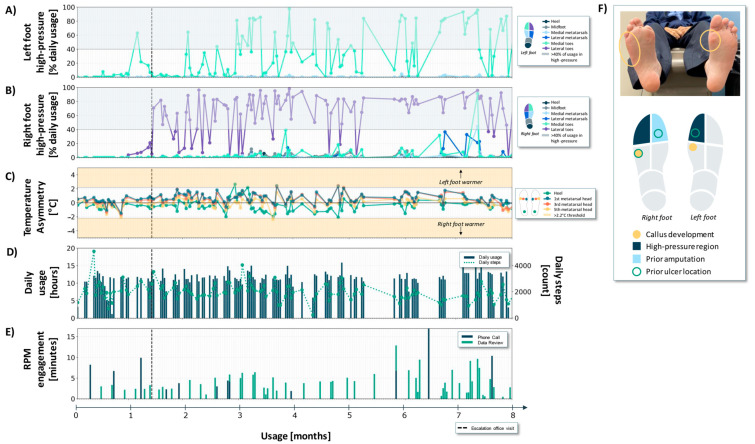
Pressure, temperature, daily usage, and RPM engagement metrics over an 8-month period for Case 2. (**A**,**B**) High-pressure states, expressed as a percentage of daily usage, for the different regions of the left and right foot, respectively. Any regions falling in the blue-shaded area were in a high-pressure state for more than 40% of the usage time. (**C**) Temperature asymmetries. Data points in the upper and lower yellow region indicate one foot is at least 2.2 °C warmer than the other. (**D**) Daily usage and step count. (**E**) RPM engagement phone calls and data review. (**F**) Photographs of the plantar surface of the patient’s feet alongside a foot map summarizing high-pressure regions, areas of callus development seen in the clinic, and prior amputations and ulcer locations.

During the 8-month usage period, the patient wore the sensory insoles for an average of 11.4 (±2.4) h per day with an average step count of 1993 (±639) steps (Figure 4D).

During a clinic visit that occurred early in the study window (black dashed vertical line in Figure 4A–E), the patient noted receiving biofeedback from the digital display warning of sustained high plantar pressures. The patient presented with a pre-ulcerative, erythematous, callused area on the plantar surface of the right fifth metatarsal head and lateral foot, as well as callus under the left first metatarsal head (Figure 4F).

While a limited amount of sensory insole data was available prior to this clinical presentation, it is posited that the observed plantar tissue damage is consistent with compensatory loading in response to the right first toe amputation [27]. Consequently, the patient shifts pressure away from the right medial foot towards the lateral side of the right foot and medial side of the left foot.

During the clinic visit, a dancer pad insole modification was placed underneath the left first metatarsal head to offload and redistribute pressure directly under that area. Additionally, a lateral post was added to the right insole to help redistribute elevated pressures from the right lateral foot to the right medial foot, away from the callused right fifth metatarsal head.

Following the clinic visit, the patient had high-pressure at the left medial toes and right lateral toes (Figure 4A,B). A total of 44 high-pressure flags throughout the 8-month window prompted 71 engagements (14 unique phone calls, 57 unique data review sessions) from the RPM nurse.

The patient did not generate many temperature asymmetries, despite the erythematous nature of the lesion on the right fifth metatarsal head. Contralateral temperature differences only exceeded the 2.2 °C threshold on five non-consecutive days during the usage window (Figure 3C) and none that preceded their in-office assessment.

### 3.3. Case 3

**Figure 5:**
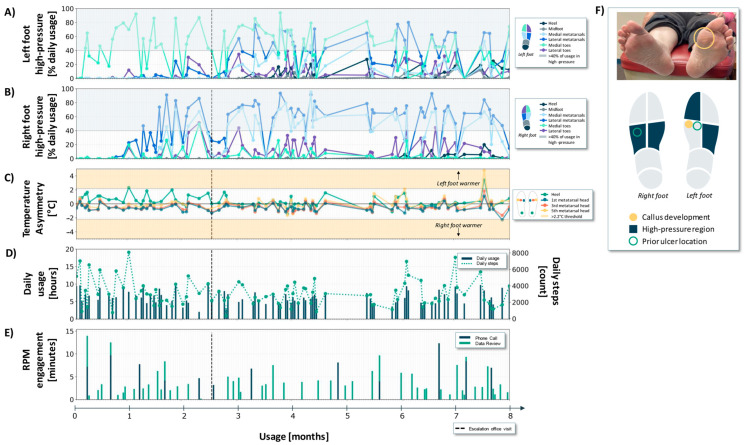
Pressure, temperature, daily usage, and RPM engagement metrics over an 8-month period for Case 3. (**A**,**B**) High-pressure states, expressed as a percentage of daily usage, for the different regions of the left and right foot, respectively. Any regions falling in the blue-shaded area were in a high-pressure state for more than 40% of the usage time. (**C**) Temperature asymmetries. Data points in the upper and lower yellow region indicate that one foot is at least 2.2 °C warmer than the other. (**D**) Daily usage and step count. (**E**) RPM engagement phone calls and data review. (**F**) Photographs of the plantar surface of the patient’s feet alongside a foot map summarizing high-pressure regions, areas of callus development seen in the clinic, and prior ulcer locations.

Case 3 is a 75-year-old female with a history of type 2 diabetes, peripheral neuropathy, and prior DFU on the right fourth metatarsal head as well as the left second metatarsal head (Figure 5F). The patient had a history of chronic, recurrent DFUs (presenting approximately one to two times per year for the last 4 years) and non-reducible hammertoes on both the left and right foot. The patient’s right and left foot are similar in shape and biomechanical deformity. The combination of these foot flexion deformities and neuropathy results in poor balance.

During the 8-month usage period, the patient wore the sensory insoles for an average of 6.2 (± 1.8) h per day. The patient showed consistent usage and a relatively high average step count of 3104 (± 1628) steps (Figure 5D). A gap in usage around month 5 is a consequence of the patient undergoing surgery unrelated to their feet.

The patient generated 46 elevated pressure flags in the 8-month window, primarily on the left medial toes. Elevated pressures were also generated on the right medial foot and bilateral lateral foot regions (Figure 5A,B). Based on the pressure flags generated, the RPM nurse escalated the patient to their treating clinician for a clinic visit (black dashed vertical line in Figure 5A–E). During that visit, the patient presented with a callus at the plantar surface of the left first metatarsal head (Figure 5F). This callus was debrided by the clinician during the visit. No structural interventions were provided, as the patient’s foot deformities could not be easily addressed through insole modifications or non-surgical offloading mechanisms, nor was there a clear indication for surgery.

Following the clinic visit, the patient continued to generate elevated pressures in the left and right metatarsals (Figure 5A,B, months ~2.5–8). During this period, repeated high-pressure events prompted 33 RPM engagements with dashboard data and seven patient phone calls.

No significant temperature asymmetries were generated in the 8-month usage window. Contralateral temperature differences only exceeded the 2.2 °C threshold on four non-consecutive days (Figure 5C).

## 4. Discussion

Approximately 40% of DFUs recur within one year of ulcer healing [5] and average recurrence rates at 6 months are estimated to be 30% [28,29,30]. Three illustrative patients with a history of recurrent plantar foot ulcers developed no ulcers while utilizing a multi-faceted digital health solution for an 8-month monitoring window. Through the combination of multimodal sensing, dynamic patient-facing biofeedback, RPM review of data trends of concern, and the human touch provided by RPM engagement, a holistic prevention system is established. As of the time of writing, all three patients are ongoing users of the system, and none have re-ulcerated. For these patients, this multi-faceted system of care appears to have successfully disrupted their chronic ulcer recurrence cycle.

### 4.1. Measuring and Managing Plantar Pressure Offloading

To the authors’ knowledge, this case series is the first study to demonstrate that remote plantar pressure monitoring is a valuable tool for the ongoing measurement and management of plantar pressure offloading adherence (see IWDGF guideline *“Adherence to appropriate footwear, including custom-made insoles, orthotic interventions, pressure-relieving interventions”*, Table 1). While the results from a randomized controlled trial demonstrated patient-directed pressure offloading feedback to be effective in reducing DFU recurrence [17], the present study extends on this work by incorporating plantar pressure monitoring into an RPM system of care. Leveraging continuous plantar pressure monitoring for multiple data-drive action pathways offers numerous benefits.

First, the identification of plantar pressure data trends of concern directly informed offloading treatment strategies. For example, in Case 1, a consistent trend of lateral left foot pressure measured by the sensory insole, along with clinical presentation of callus and cracking on the plantar surface, motivated an additive insole modification (left lateral post). The intention of this data-driven modification was to redistribute pressure away from the lateral left foot to mitigate sustained high-pressure and DFU risk at the callus site.

Second, continuous monitoring of sustained high plantar pressures offered insight into pressure offloading following additive insole interventions. For example, in Case 1, ongoing pressure monitoring revealed a trend towards a gradual change in sustained high-pressures following the addition of a lateral post, consistent with an expected, if delayed, impact in gait retraining. Alternatively, in Case 2, continuous monitoring revealed persistent regions of sustained high-pressures following the insole modifications, suggesting the additive insole modification did not impact the sustained high plantar pressure as expected. Motor learning of gait modifications is a complex process that can take significant time [31] and it is plausible that further gait modifications and changes in elevated and sustained pressures could have manifested beyond the monitoring window of this study. Given that approximately 50% of wounds recur on the contralateral foot, and most remaining recurrences are at a different location on the same foot [32], continuous plantar pressure monitoring further serves to ensure that offloading treatments do not unintentionally introduce pressure overload risk at anatomical sites distant from the previous wound.

Third, in all three cases, the pressure-offloading education delivered during the frequent RPM engagements was informed by data trends of concern (Figure 3, Figure 4 and Figure 5E) and supplemented patient-facing biofeedback (pressure offloading cues). It is well-established that offloading adherence is especially important for ulcer healing and recurrence prevention [17,33]. The cadence and volume of the RPM interactions (Figure 3, Figure 4 and Figure 5E) are tuned to concerning physiologic data trends that are leading indicators of tissue injury, or gaps in adherence that may be indicative of reduced participation in self-management practices. While we did not quantify the impact of RPM engagement on offloading behavior, we speculate that the frequent reinforcement empowered patients with a high level of self-management (see IWDGF guidelines, *“Adherence … pressure-relieving interventions”*, Table 1).

### 4.2. Multi-Faceted Digital Health Solutions Enable and Reinforce Integrative Foot Care Guidelines

Multi-faceted digital health solutions offer potential for supporting the spectrum of care required for complex conditions such as diabetic foot disease [24]. No single strategy in isolation supports all guidelines for the prevention and management of DFUs (Table 1). Multi-faceted digital health solutions offer key advantages for enabling and reinforcing integrative foot care guidelines.

First, expanding to multimodal sensing (rather than monitoring/actioning on a single physiologic signal) supports the monitoring of warning indicators at several stages throughout the DFU pathogenesis pathway [25]. Despite pressure overload and callus formation playing a central role in DFU pathogenesis [2], many digital health solutions designed for DFU risk reduction focus only on plantar temperature monitoring [19,22,23]. Notably, in the three cases presented, contralateral temperature asymmetries remained in the acceptable range (i.e., no consecutive days with greater than 2.2 °C asymmetry) throughout the monitoring period (Figure 3, Figure 4 and Figure 5). Conversely, at the stages of DFU development for these cases, plantar pressure monitoring provided warning indicators that supported the management of pressure offloading. It is likely that with continued monitoring of these cases (>8 months), as well as other future patient cases, DFU pathogenesis may progress differently, and as such, temperature monitoring, or a combination of pressure and temperature monitoring is likely vital. Multimodal sensing aligns with the dynamic and time-varying nature of DFU pathogenesis.

Second, multimodal sensing provides redundancy in monitoring when one or more physiological signals are confounded by underlying conditions or external factors. For example, monitoring in the pressure domain may serve as an important adjunct to temperature monitoring regimes, which may be confounded by comorbidities common in individuals with diabetes. Patient immunocompromise may impact the sensitivity of established plantar temperature asymmetry thresholds, while vascular disease may impact the specificity of these measurements [21,34]. Case 2 highlights a patient who is post renal transplant because of ESRD from diabetic nephropathy. Despite their pre-ulcerative lesion having some erythematous (redness) attributes, it did not appear to manifest as an insole-based plantar temperature asymmetry. The capacity of such patients to generate temperature differentials in the foot related to pre-ulcerative inflammation remains understudied. Multimodal sensing offers flexibility and enables care to be personalized to a patient’s health profile.

Finally, multi-faceted digital health technologies can offer accessible opportunities to promote patient engagement and adherence to their foot health management plan. Case 3 illustrates a patient at a high risk of recurrence with limited clinical interventions available to guard against recurrent DFUs. In this case, plantar pressure data trends measured by the sensory insoles, alongside a clinical presentation of a callus, informed clinician intervention (debridement). However, additional offloading mechanisms, such as insole modifications, were not indicated in view of the patient’s existing foot deformities. Ongoing plantar pressure monitoring, patient-directed active pressure offloading biofeedback, and periodic and convenient interactions with a remote healthcare professional trained in diabetic foot management provided multi-layer care to a patient with few non-surgical treatment options. Multi-faceted digital health technologies offer patients and clinicians alternative and comprehensive treatment plans to fit the patient’s medical and lifestyle needs.

## 5. Limitations

### 5.1. Study Design Limitations

This case series is limited by its sample size and non-consecutive nature. It is exploratory, and conclusive causal inferences should not be drawn. Larger cohort studies and randomized clinical trials are warranted to explore the benefits of multi-faceted digital health solutions in proactively detecting pre-ulcerative indications and preventing escalations to more serious foot complications.

### 5.2. Technology Limitations

The sensory insoles used in these case examples only capture plantar physiological data while the patient is wearing the device, and thus do not detect any foot risks that may arise while not being worn. Additionally, the sensory insoles have pressure arrays that are configured and optimized for the detection of sustained high-pressure over time to limit adverse pressure-related events. As such, it would not necessarily be expected to see all impacts of pressure redistribution manifest in the data. Extended pressure monitoring configurations may support valuable evaluations such as the specific pressure redistributions achieved with a given insole modification. Finally, despite the product design choices informed by durability testing, the performance of some sensor components (e.g., mechanical components of pressure sensors) might change over time due to wear and tear to the product. Variability in calibration procedures performed at the time of production may also influence the performance of some sensors (e.g., pressure threshold calibration process).

## 6. Future Opportunities

Given the emerging nature of multi-faceted digital health programs, there are numerous exciting future research opportunities to evaluate and optimize such care strategies. Based on the learning from the patient journeys in these cases, the authors offer a (non-exhaustive) list of existing uncertainties and underexplored topics surrounding the digital health management of DFU that warrant future research.

First, the individual and combined benefits of each aspect of a multi-faceted digital health solution are unknown (e.g., sensory insole monitoring, real-time biofeedback for pressure offloading, RPM engagement, clinician involvement, and/or insole modifications). Quantifying the impact of each of these individual components may also continue to inform which feedback/action pathway(s) are best paired with each physiological signal. Similarly, the cost effectiveness of multimodal sensor-based RPM programs versus their individual components remains understudied. Although cost savings relating to DFU treatment have been shown with real-time biofeedback and remote temperature monitoring alone [20,35], the cost effectiveness of sensor-based RPM programs warrants further research.

Second, both quantitative and qualitative characterization of user adherence and the barriers to use (e.g., how aspects such as comfort, ease of use, etc., drive adherence) are essential for driving future technological innovations with the aim of further optimizing patient engagement [15]. While no formal user experience analyses were performed in this study, the patients anecdotally reported the system to be comfortable, easy to use, and appreciated the opportunity to actively engage in their foot health through the biofeedback alerts and RPM engagement.

Third, there is always an opportunity for ongoing refinement of the warning thresholds for both plantar pressure and plantar temperature monitoring to balance sensitivity and specificity. These thresholds may need to be tuned to a patient’s dynamically changing risk profile, and there may be opportunities in multimodal systems to develop warning thresholds for certain combinations of elevated physiologic parameters.

Fourth, there are infinite combinations of sensor suites and sensor configurations that can be deployed in a given digital health technology. Ongoing product research and development efforts may reveal new combinations of existing sensor suites and physiological signals, or entirely new wearable sensors that are effective for continuous DFU management.

Lastly, the emerging nature of these technologies limits the ability to systematically correlate the sensor-based data trends with certain clinical conditions and treatments. The system currently relies on the RPM nurse to communicate with the patient to gather medical, behavioral, and lifestyle context, bridge the gap between them, and decide on an action plan. Future wearable technologies might benefit from developing integrated databases that fuse sensor-based data with patient medical history. Furthermore, there are exciting opportunities to integrate monitoring technologies across multiple clinical domains, creating holistic ecosystems for managing chronic conditions.

## 7. Conclusions

This case series demonstrates the value of a multi-faceted digital health solution combining multimodal data collection, patient-facing biofeedback, and remote patient monitoring to enable and reinforce diabetic foot health management guidelines. Across three illustrative patients, continuous plantar pressure monitoring demonstrated promise for empowering both the patient and provider with information for the data-driven management of pressure offloading treatments. While most remote monitoring digital health solutions for foot ulcer prevention focus on plantar temperature monitoring, some clinical comorbidities may limit or confound the utility of plantar temperature monitoring for DFU risk, highlighting the value of capturing multiple continuous sensor-based physiological data streams.

Multi-faceted digital health solutions can naturally address many of the challenges with established diabetic foot care guidelines, motivating ongoing research to optimize and explore the benefits of such solutions. Rather than relying on a single signal, multi-modal sensing across multiple physiological domains supports the monitoring of foot health at multiple stages along the DFU pathogenesis pathway. Furthermore, digital health solutions equipped with remote patient monitoring provide new opportunities for personalizing treatments, providing periodic self-care reinforcement, and encouraging patient engagement—tools for improving patient adherence to their diabetic foot care plan. By serving as a tool to disrupt a patient’s chronic ulcer recurrence cycle, holistic digital health solutions support the broader goals of health span extension for patients living with diabetes.

## Figures and Tables

**Table 1:** The International Working Group on the Diabetic Foot (IWDGF) guidelines on the prevention and management of DFUs, challenges with adhering to these guidelines, and how multi-faceted digital health solutions overcome existing challenges to enable and reinforce these guidelines.

IWGDF Guideline [9]	Challenges	How Multi-Faceted Digital Health Solutions Enable and Reinforce Diabetic Foot Care Guidelines
Identifying the at-risk foot: examination and screening for signs and symptoms that place a patient at risk	Discontinuities in foot care due to other comorbidities or life circumstances (e.g., difficulty accessing office visits, or other social determinants) [10]. Difficulty for clinicians to personalize care and education to a patient’s lifestyle and risk profile.	Remote patient monitoring (RPM) enables continuity of care when access is a barrier. Digital health technologies provide specific data trends of concern for review by the clinical treatment team, enabling personalized, proactive management.
Regular self-exams	Difficulty performing foot self-exams due to mobility or vision limitations [7]. Limited at-home support [11].	RPM interventions in response to data trends of concern involve self-exam, if possible. Structured education with regards to self-exam importance and technique are delivered and reinforced at regular intervals.
Structured education around appropriate foot self-care	Limited retention or recall of provided medical information when not reinforced [12]. Limited opportunities to re-emphasize the self-care regimen [5].	RPM engagement enables periodic reinforcement of foot self-care best practices to maximize effect.
Self-monitoring of foot skin temperatures once daily	Difficulty performing foot self-exams due to mobility or vision limitations [7]. Difficulty recognizing the subtle early signs of a wound [13,14].	Continuous, objective temperature monitoring enabled by handheld thermometers or plantar temperature monitoring technologies; adherence is quantifiable.
Adherence to appropriate footwear, including custom-made insoles, orthotic interventions, or pressure-relieving interventions.	Insufficient adherence [15,16]. Difficulty successfully offloading plantar areas of risk.	Digital health technologies can quantify adherence to aspects of the care plan (e.g., prescription footwear or activity adherence). RPM interventions in response to decreased adherence aim to encourage patient participation in the care plan. Continuous, real-time pressure monitoring and active offloading cues enabled by plantar pressure monitoring technologies.
Treating ulcer risk factors Treatment of any pre-ulcerative signs or callus on the foot	Difficulty recognizing the subtle early signs of a wound [13,14].	Multimodal sensing and RPM intervention may help with earlier detection of pre-ulcerative signs and risk factors, escalating those patients for clinical assessment and treatment.
Foot and mobility related exercises aimed to reduce DFU risk factors, including communication around safe activity levels	Insufficient information on patient activity and its impact on patient risk.	Activity quantification through activity monitoring technologies aids in the management of activity prescription and counselling regarding appropriate activity modifications.

## Data Availability

Data are contained within the article.
